# Severe Hemorrhage from the Umbilical Cord at Birth: A Preventable Cause of Neonatal Shock

**DOI:** 10.1155/2013/317627

**Published:** 2013-11-04

**Authors:** Neetu Singh, Gautham Suresh

**Affiliations:** Department of Pediatrics, Division of Neonatology, Dartmouth Hitchcock Medical Center, 1 Medical Center Drive, Rubin 529, Lebanon, NH 03756, USA

## Abstract

Posthemorrhagic anemia is a rare but important cause of anemia in neonates, second only to hemolytic anemia of newborn. Most cases of posthemorrhagic anemia are reported from fetomaternal hemorrhage or umbilical cord accidents in utero. This case report describes a preterm infant who developed severe anemia and shock immediately after delivery related to an acute hemorrhage through patent umbilical cord vessels secondary to a tear in the umbilical cord at the site of cord clamping. We believe that umbilical cord bleeding from errors in cord clamping could be an important cause of acute blood loss in the delivery room and that it may result in significant clinical morbidity, especially in extremely premature infants.

## 1. Introduction

Posthemorrhagic anemia in the newborn can result from antenatal, intrapartum, or postnatal causes. However, there is a paucity of literature on causes of neonatal blood loss during or immediately after delivery, particularly those related to bleeding from the umbilical cord at the time of birth. This preventable cause of acute postnatal blood loss has not been emphasized in the literature before and may be more common than previously recognized. 

## 2. Case Presentation

A 24-week-gestation infant, a product of a dichorionic-diamniotic twin pregnancy, was born by spontaneous vaginal delivery after breech presentation to a 29-year-old G3P0 female. The pregnancy was complicated by a history of maternal substance abuse (marijuana) and premature prolonged rupture of membranes. The delivery was attended by the neonatal resuscitation team, and resuscitation was performed in the standard manner. A polyethylene wrap was placed over the baby, and he was intubated within few minutes for poor respiratory effort. The Apgar scores were 4, 6, and 7 at 1, 5, and 10 minutes of life, respectively. At five minutes of life, he was noted to be pale and cold. The nurse observed a pool of blood underneath the baby's back upon lifting the polyethylene wrap. Examination of the umbilical cord revealed that the cord clamp was tightly closed and had not come loose (our first suspicion); however, there was a tear in the umbilical cord between the clamp and the baby, from which the baby had lost a large amount of blood. 

An umbilical venous catheter was urgently placed, and 10 mL/kg of uncrossed type O, Rh negative packed red blood cells was transfused followed by a second 15 mL/kg aliquot an hour later. An arterial blood gas obtained at 30 minutes of life and before the blood transfusion revealed severe metabolic acidosis with a pH of 7.03, a PaO_2_ of 54 mmHg, a PaCO_2_ of 41 mmHg, and a base deficit of −20 mmol/L. 

The initial pretransfusion hemoglobin obtained within 30 minutes of delivery was 8.8 g/dL with a hematocrit of 28.7%. We estimated that this infant had lost about 15% to 20% of his blood volume (assuming the baby's actual hematocrit to be same as his twin brother). After two blood transfusions (a total volume of 25 mL/kg), the hemoglobin increased to 16 g/dL.

This infant had an immediate postresuscitation course complicated by severe anemia and disseminated intravascular coagulation, requiring multiple transfusions of blood products. He also suffered from acute renal failure and a grade IV intraventricular hemorrhage with midline shift, in addition to his underlying severe respiratory distress syndrome. Given his status of critical illness with multisystem organ dysfunction, persistent severe metabolic acidosis, and his failure to improve despite full-scale intensive treatment, the decision was made to discontinue intensive care after multiple discussions with the infant's family. He passed away soon thereafter.

## 3. Discussion

Extremely preterm infants are in a fragile condition at birth, and hemorrhagic anemia and shock due to blood loss at birth can further adversely impact their hospital course and final outcome. In this infant, the significant blood loss at birth from a tear in the umbilical cord led to shock and severe metabolic acidosis, necessitating emergency blood transfusion in the delivery room in addition to standard resuscitation measures. Such umbilical cord hemorrhage can also occur if the umbilical cord clamp placed prior to cutting of the cord becomes loosened or does not occlude one or more of the umbilical vessels (partial clamping of the cord). 

The incidence and consequences of umbilical cord hemorrhage immediately after birth from errors in cord clamping are not well addressed either in the literature or in current clinical practice guidelines. A review of the literature yielded just one case series from the 1950s that described 36 cases of umbilical cord bleeding within the first 48 hours of life over a period of 15 years [1]. This paper emphasized that there are clearly rare instances when natural mechanisms of hemostasis in the umbilical cord fail, leading to massive blood loss from the cord and adverse outcomes, including death. In another study [2], umbilical cord hemorrhage in a developing country was noted to occur in about 9% of home births. The consequences of massive blood loss in newborns are grave. When the total volume is reduced by 10%–15%, the newborn develops abnormalities in both the peripheral and central circulatory responses [3]. The impact is magnified in preterm infants due to their inability to adequately compensate for acute loss of blood volume and the complications from severe hypotension and shock such as intraventricular hemorrhage and periventricular leukomalacia [4]. The risk of such an event occurring in the extremely premature infant is further increased by the inherent urgency to hand off the infant to the waiting neonatal team at delivery. Finally, the impact is further magnified by delays in detecting these events from our current resuscitation practices such as covering the extremely premature infant with occlusive plastic wrap to prevent hypothermia and elective intubation for prophylactic surfactant administration. During home births in developing countries, poor ambient light may also reduce visibility and contribute to delayed detection of umbilical cord hemorrhage. 

Neonatal resuscitation is usually performed according to a standard algorithm promulgated by the Neonatal Resuscitation Program, which emphasizes prevention of heat loss and assessment and management of respiration and of heart rate. The algorithm does not include a specific recommendation to inspect the baby's umbilical cord to detect bleeding. From our experience, we feel that every infant immediately after delivery should, in addition to the evaluation recommended by the NRP, undergo a quick inspection of the umbilical cord and cord clamp to detect cord hemorrhage and stop it before excessive blood loss occurs. Fortunately, these events are preventable if we can increase the awareness of the possibility of acute blood loss from errors in cord clamping. To ensure early detection of all such hemorrhages, we strongly feel that a quick cord inspection should be a routine component of the initial assessment during resuscitation of any newborn baby. 

## Abbreviations

IVH: Intraventricular hemorrhage
PVL: Periventricular leukomalacia
NRP: Neonatal Resuscitation Program.
